# A 10-year clinical outcome of radiotherapy as an adjuvant or definitive treatment for primary tracheal adenoid cystic carcinoma

**DOI:** 10.1186/s13014-017-0933-6

**Published:** 2017-12-04

**Authors:** Hyoung Uk Je, Si Yeol Song, Dong Kwan Kim, Yong-Hee Kim, Seong-Yun Jeong, Geum Mun Back, Wonsik Choi, Su Ssan Kim, Seung-Il Park, Eun Kyung Choi

**Affiliations:** 1 Department of Radiation Oncology, Ulsan University Hospital, University of Ulsan College of Medicine, Ulsan, South Korea; 2 Department of Radiation Oncology, Asan Medical Center, University of Ulsan College of Medicine, 88, Olympic-ro 43-gil, Songpa-gu, Seoul, 05505 South Korea; 3 Department of Thoracic Surgery, Asan Medical Center, University of Ulsan College of Medicine, Seoul, South Korea; 4 Asan institute for Life Science, Asan Medical Center, University of Ulsan College of Medicine, Seoul, South Korea; 5 Department of Medical Health Science, Kangwon National University Graduate School, Chuncheon, South Korea; 6 Department of Radiation Oncology, Gangneung Asan Hospital, University of Ulsan College of Medicine, Gangneung, South Korea

**Keywords:** Tracheal cancer, Adenoid cystic carcinoma, ACC, Radiotherapy, Unresectable, Survival

## Abstract

**Background:**

To evaluate the role of radiotherapy (RT) as an adjuvant or definitive treatment in primary tracheal adenoid cystic carcinoma (ACC) for local tumor control and survival.

**Methods:**

A retrospective chart review was performed in 22 patients treated with adjuvant or definitive RT for primary tracheal ACC at a single center between November 1994 and December 2008.

**Results:**

Thirteen and 9 patients received adjuvant and definitive RT, respectively. Microscopic residual disease after surgery was pathologically reported in 11 patients. The median RT dose was 59.4 Gy for adjuvant and 74.4 Gy for definitive RT. The overall response rate for definitive RT was 77.8%. Six patients in the definitive RT group exhibited local progression (LP), whereas 14 patients in both groups exhibited distant metastasis. The most common recurrence site in cases of treatment failure was the lung parenchyma. The median follow-up duration was 123 months, and the 10-year overall survival (OS) rate was 54.2%. Although LP was the most common cause of death (4 patients), two-thirds of the patients treated with definitive RT lived for >5 years. The 5-year and 10-year LP-free survival (LPFS) rates in the definitive RT group were 66.7 and 26.7%, respectively. Patients with higher RT dose by brachytherapy boost had good 5-year OS, 83.3%, and showed no local progression till 5-years. Most of the RT-induced side-effects were mild and tolerable, but 2 patients died of tracheal stenosis without any tumor recurrence.

**Conclusions:**

Adjuvant RT may be suitable for controlling microscopic residual disease, whereas definitive RT may yield appropriate long-term survival in >50% patients with unresectable tracheal ACC. Dose escalation should be considered to warrant long-term survival in definitive RT.

## Background

Tracheal cancer is a rare neoplasm that accounts for only 2% of upper airway tumors [1]. The incidence of primary tracheal tumors is <0.2 per 100,000 persons per year in the United States [2]. Squamous cell carcinoma (SCC) is the most common histological type of tracheal malignancy, followed by adenoid cystic carcinoma (ACC) [3, 4]. Tracheal ACC was first reported by Morgagni in 1762. ACC of the trachea and bronchi arises from glands in the tracheobronchial mucosa. ACC is characterized by slow growth, and distant metastasis has been reported as late as 25 years after diagnosis [5–7].

Surgery is considered as the treatment of choice for ACC, and most articles have focused on the surgical outcome. Radiotherapy (RT) was traditionally used as an adjuvant treatment for controlling microscopic disease or as a salvage treatment for unresectable disease; however, the exact role of RT remains unclear due to the rarity of such reports. Although a few studies have described the use of surgery followed by adjuvant therapy, only a few factors related to RT planning or dose have been described [7–9]. In particular, for definitive RT, additional information should be provided, as most retrospective studies thus far have analyzed a very small number of patients, have used a classical RT technique or irradiated a relatively lower RT dose [6, 8, 10, 11]. The most recently available data on modern RT techniques are described in case reports, and these provide promising results for RT of ACC of the trachea [12–14].

In the present study, we aimed to evaluate the role of RT with modern techniques as adjuvant treatment after surgical resection or as definitive treatment in inoperable settings for primary tracheal ACC for local tumor control and survival.

## Methods

Between November 1994 and December 2008, a total of 25 patients were treated with RT for pathologically confirmed primary ACC of the cervical or upper thoracic airway in our institution. We retrospectively reviewed the medical records and the study was approved by the institutional review board of Asan Medical Center, Seoul, Korea (AMC-IRB, 2012–0470). Tumor location was assessed by using a bronchoscope or computed tomography (CT), and cases with tumors located in the trachea and carina with/without main bronchus involvement were included in the study. The epicenters of primary tumor in all patients were trachea. We excluded patients with a distant metastasis prior to treatment. However, 1 patient with single lung metastasis at the initial diagnosis was included in this study, because the metastatic lesion had been successfully treated with stereotactic body radiotherapy and complete remission was achieved in that case.

The tracheal tumor stage was determined according to the staging system for primary tracheal malignancies proposed by Bhattacharyya et al [4]. We measured tumor size by using a fiber-optic bronchoscope or CT scan.

The eligibility for surgical excision was determined via a multidisciplinary conference or clinic, after reviewing the results of diagnostic studies and the patient’s condition. A dedicated thoracic surgeon determined the surgical approach and extent.

Adjuvant RT was usually initiated 4–6 weeks after surgery, and the standard dose was 50.4 Gy for 28 fractions. A boost dose was provided if the tumor had a surgical margin. Approximately 60–66 Gy of conventional fractionation was the standard dose for definitive RT, although brachytherapy (BT) was occasionally adopted for a local boost. In all cases of RT, a linear accelerator with three-dimensional conformal RT or intensity-modulated RT (IMRT) was used, and an iridium-192 high-dose BT system was employed. BT was conducted at 1 cm from the iridium source, and a boost dose of 15 Gy or 21 Gy with 5–7 Gy per fraction was used. The initial irradiation field encompassed the tumor or tumor bed as well as the adjacent mediastinal or supraclavicular lymph nodes in most patients. Clinical target volume (CTV) was usually expanded 3 cm in longitudinal from tumor bed or primary tumor and adjacent lymph node stations were included it at designated level. Planning target volume (PTV) was generally expanded 1 cm in both longitudinal and axial from the CTV. Dose constraints for organ at risk (OAR) like esophagus, lung or other organs were not absolutely determined in this study, while we tried not to produce hot-spot at esophagus due to the proximity to PTV and institutional dose constraint for whole lung was V20 < 30%.

In the definitive RT group, we judged tumor response according to the Response Evaluation Criteria in Solid Tumors (RECIST) criteria [15]. Response evaluation and routine follow-up was performed by CT images and bronchoscopy with/without biopsy was added to confirm residual tumor after definitive RT. Positron emission tomography (PET) was adopted in only a half patient, because it was not introduced in the early phase of this enrollment. PET was additionally used to confirm local progression or distant metastasis in some cases, but not routinely acquired. The initial response of RT was assessed at 1 or 2 months after RT. We used the Radiation Therapy Oncology Group (RTOG) toxicity grading system to score acute and chronic complications. Patients visited the outpatient clinic at 3-month intervals during the initial 2 years and at 6-month intervals thereafter.

The survival rate was calculated using the Kaplan-Meier survival analysis method. Survival curves were compared using the log-rank test and odds ratio from cox-regression analysis was calculated for multivariate analysis. All analyses were based on a two-sided test for significance (0.05), and were conducted using the SPSS Statistics 21.0 software (SPSS Inc., Chicago, IL).

## Results

### Patient and treatment characteristics

A total of 22 patients were analyzed; three patients were excluded due to coincident multiple lung metastasis or because they were lost to follow-up immediately after treatment, with no evidence of progression or toxicity. Patient characteristics are presented in Table 1. Fourteen (63.6%) patients were female. The most common presenting symptom was dyspnea. In many cases, symptoms of airway obstruction, such as dyspnea or wheezing, led to a misdiagnosis of asthma, which delayed the diagnosis of ACC.

**Table 1:** Patient characteristics

	Number of patients (%)	
	PORT	Definitive RT
Total number of patients	13	9
Median age, years (range)	40 (27–69)	44 (37–68)
Gender		
Male	3 (23)	5 (56)
Female	10 (67)	4 (44)
Initial presenting symptom		
Dyspnea	9 (69)	7 (78)
Cough	1 (8)	2 (22)
Hoarseness	2 (15)	
None	1 (8)	
Location of the tumor		
Upper half, cervical	5 (38)	3 (33)
Lower half, thoracic	3 (24)	4 (45)
Carina involving main bronchus	5 (38)	2 (22)
Clinical T stage		
T1	6 (46)	
T2	7 (54)	8 (89)
T4		1 (11)
Clinical N stage		
N0	13 (100)	9 (100)
Clinical M stage		
M0	13 (100)	8 (89)
M1		1 (11)

*PORT* post-operative radiotherapy, *RT* radiotherapy

The treatment characteristics are summarized in Table 2. Thirteen patients underwent surgery, and the resection margin status was R1 in 11 patients (84.6%). These 13 patients received adjuvant RT and the other 9 patients received definitive RT. The fraction size of external beam radiotherapy (EBRT) ranged from 1.8 Gy to 2.2 Gy, and 3 or 4 sessions of 5–7 Gy BT were conducted for a local boost after EBRT in some cases. Two patients in definitive RT group received IMRT without BT boost. Only 1 patient received concurrent chemotherapy with gemcitabine plus cisplatin.

**Table 2:** Treatment characteristics

	No. (%)
*Surgery*	13
Type	
Tracheal resection	6 (46.2)
Laryngotracheal resection	1 (7.7)
Bronchial resection	1 (7.7)
Pneumonectomy	4 (30.7)
Endobronchial resection	1 (7.7)
Resection margin status	
R0	2 (15.4)
R1	11 (84.6)
*Radiotherapy*	22
Type	
PORT	13 (59.1)
Definitive RT	8 (36.4)
Definitive CCRT	1 (4.5)
Technique	
EBRT alone	16 (72.7)
EBRT and brachytherapy boost	6 (27.3)
Median dose (Gy)	59.8 (range, 50.4–80)
PORT	59.4 (range, 50.4–66)
Definitive RT	74.4 (range, 66–80)
Radiotherapy field	
Trachea only	1 (4.5)
Trachea with regional lymph node	21 (95.5)

*PORT* post-operative radiotherapy, *CCRT* concurrent chemo-radiotherapy

### Tumor response and pattern of failure

One patient exhibited complete remission (CR), and showed only fibrotic residual tissue on biopsy, after definitive RT. None of the patients exhibited progressive disease during the initial response evaluation. The overall response rate, including CR or partial remission (PR), in 9 patients with an inoperable tracheal tumor was 77.8% (Table 3).

**Table 3:** Details of the patients who were treated with definitive radiotherapy

Sex	Age	T	Size (cm)	RT dose	BT boost	RESP	LP	RT- LP (mo)	DM	RT-DM (mo)	Cause of Death	OS (mo)
F	61	2	4	70.8	+	CR	–		–		Stenosis	172
M	48	2	6	70.2		PR	+	36	–		LP	107
M	44	2	6	79.8	+	PR	+	104	+	28	LP	107
M	68	4	5	74.4	+	PR	–		+	18	DM	51
M	36	2	4	74.4	+	PR	–		+	85	Other	232
M	37	2	6	80.0	+	PR	+	77	+	24	DM	115
F	37	2	5	79.8	+	SD	+	87	+	29	LP	87
F	66	2	7	66.0		SD	+	17	+	17	LP	32
F	43	2	7	66.0		PR	+	21	+	21	DM	26

*T* T-stage, *RT* radiotherapy, *BT* brachytherapy, *RESP* initial response after RT, *LP* local progression, *DM* distant metastasis, *RT-LP* time interval from RT to LP, *RT-DM* time interval from RT to DM, *OS* overall survival, *mo* months

Six (27.3%) patients developed local progression (LP) during the follow-up period, although LP developed at the initial nidus only in 3 cases. LP developed only in patients treated with definitive RT without surgery. LP was pathologically confirmed in 3 patients experiencing early LP, while progression in the other patients was judged by gradual tumor enlargement on serial CT image. A total of 14 (63.6%) patients developed distant metastasis, and the most common site was lung parenchyma. The mean duration to lung metastasis in 12 patients was 88.3 months. According to the first site of failure, the main pattern of failure was distant metastasis (Table 4).

**Table 4:** Treatment results for all patients

	All patients	PORT	Definitive RT	*p* value
No. of patients	22	13	9	
Local recurrence	6 (27%)	0	6 (67%)	
Distant metastasis	14 (64%)	7 (54%)	7 (78%)	
First site of recurrence				
Local	1 (5%)	0	1 (11%)	
Distant	12 (55%)	7 (54%)	5 (56%)	
Local & distant	2 (9%)	0	2 (22%)	
5-yr. LPFS (%)	85.7	100.0	66.7	<.01
10-yr. LPFS (%)	70.2	100.0	26.7	
5-yr. DMFS (%)	52.6	67.7	33.3	.209
10-yr. DMFS (%)	33.5	42.3	22.2	
5-yr. DFS (%)	47.8	67.7	22.2	.054
10-yr. DFS (%)	28.7	42.3	11.1	
5-yr. OS (%)	81.8	92.3	66.7	.058
10-yr. OS (%)	54.2	76.9	22.2	

*RT* radiotherapy, *PORT* post-operative radiotherapy, *LPFS* local progression free survival, *DFS* disease free survival, *OS* overall survival

### Survival

The median follow-up duration was 123 months (10.2 years; range, 13–232 months). The 5-year and 10-year overall survival (OS) rates were 81.8 and 54.2%, respectively. Fifteen (68.2%) patients died during the follow-up period. The cause of death was LP in 4, lung metastasis in 2, peritoneal seeding in 2, other metastasis in 3, tracheal stenosis in 2, and no relation to the disease in 2 cases. The 5-year and 10-year disease-free survival (DFS) rates were 47.8 and 28.7%, respectively. None of the patients developed LP or distant metastasis after 10 years. The 5-year LP-free survival (LPFS) rate was 100% in the postoperative RT (PORT) group and 66.7% in the definitive RT group (*p* < 0.01). Three patients developed LP over 5 years after RT, and the 10-year LPFS rate decreased to 26.7% after definitive RT. The mean LPFS in all 22 patients was 122 months, whereas that in the definitive RT group was 57 months (Table 4).

### Prognostic factor

**Fig. 1:**
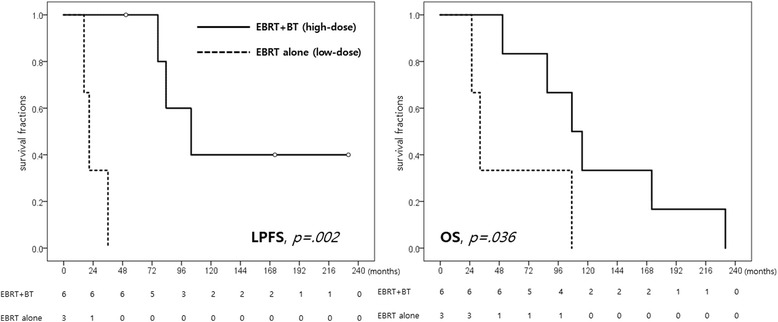
Radiotherapy dose and survival in definitive RT (LPFS: Local progression free survival, OS: Overall survival)

Initial local tumor stage (T1 vs. T2–4) was a unique prognostic factor for OS (*p* = 0.045), whereas surgery had a significance for LPFS (*p* < 0.01) in univariate analysis. Surgery tended to prolong OS, but it couldn’t reach statistical significance (*p* = 0.058) However, no factor could reach statistical significance for OS or LPFS in multivariate analysis, but odds ratio of PORT for LPFS was very low value, 0.005, which might mean the decreased risk of local progression (Table 5). Gender or location of primary tumor had no effect on treatment results. In sub-analysis with only definitive RT group, intensified RT dose with BT boost was a statistically significant factor for OS (*p* = 0.036) and LFPS (*p* = 0.002) (Table 6) (Fig. 1).

**Table 5:** Prognostic factors for survival; univariate and multivariate analysis

	Description	Uni-variate		Multi-variate					
		*p-value*		Odds ratio		95% CI		*p-value*	
		OS	LPFS	OS	LPFS	OS	LPFS	OS	LPFS
Gender	Male vs. Female	.455	.511						
Location	Cervical vs. Thoracic	.910	.986						
T stage	T1 vs. T2–4	.045	.075	3.144	1.014	0.566–17.469	*	.190	.999
Surgery	Definitive RT vs. PORT	.058	.001	0.628	0.005	0.185–2.132	*	.456	.338

*CI* confidence interval, *OS* overall survival, *LPFS* local-progression free survival, *PORT* post-operative RT, * two wide range: 0-maximal

**Table 6:** Radiotherapy dose and survival in definitive RT (*n* = 9)

	EBRT alone (low-dose)	EBRT + BT boost (high-dose)	*p-value*
No. of patients	3	6	
RT dose (Gy)			
Median	66.0	77.1	
Range	66.0–70.2	70.8–80.0	
5-yr. LPFS (%)	0.0	100.0	.002
5-yr. OS (%)	33.3	83.3	.036

*RT*: radiotherapy, *EBRT* external-beam RT, *BT* brachytherapy, *LPFS* local-progression free survival, *OS* overall survival

### Treatment-related complications

The most frequent complaint during RT was odynophagia, although the condition could be tolerated without narcotic analgesics in most patients. Three patients developed grade 3 esophagitis during RT, but there was no esophageal stenosis after treatment. Nine patients developed grade 2 or lower radiation-induced lung fibrosis.

The major concern after treatment was tracheal stenosis (2 patients). Tracheal stenosis without any evidence of recurrence developed 1 year after definitive RT in 1 case. This patient lived for 13 years after the diagnosis of stenosis, but needed the implantation of a tracheal stent. She finally died of tracheal infection around the stent. Another patient received PORT after surgery with permanent tracheostomy. She complained of dyspnea at 2 months after RT, but did not exhibit any recurrence. Nevertheless, she suffered from the narrowing of the tracheostomy site due to the gradual increase in granulation tissue, and died of abrupt respiratory failure after 1 year.

## Discussion

Adenoid cystic carcinoma originally arises from the salivary glands and is a slowly progressing malignancy, with late metastasis to the lung, bone, and brain. The most common site in the airway is the trachea and the prognosis for ACC is better than that for SCC tracheal cancer [16, 17]. A few retrospective studies on the outcome of RT for tracheal ACC are summarized in Table 7. The studies mostly reported good 5-year OS of >70%; however, the results could not be directly compared with those of the current study, due to the lack of a definite consensus about the staging system for tracheal tumor and the difference in the treatment modalities.

**Table 7:** Historical reports of treatment for ACC of the trachea

Author	Year	Pt. No.	Treatment	RT dose (Gy)	5-yr. OS (%)	Mean OS (months)
Grillo [7]	1990	80	S	–	–	39
			S + R	NA	–	107
Gelder [8]	1993	34	S(9), S + R(10) R(11), C(4)	NA	80	–
Maziak [6]	1996	38	S(6), S + R(26)	NA	79	87
			R(6)	50–75	–	74
Regnard [9]	1996	65	S(37), S + R(28)	NA	73	
Prommegger [20]	1998	16	S(16)		79	–
Bhattacharyya [4]	2004	19	NA	NA	78	–
Honings [21]	2010	108	S(19), S + R(89)	54	78	–
Lee [22]	2011	29	S ± R(17)	54	100	143
			R(12)	60	54	61
*This study*	2017	22	S + R(13)	50	92	136
			R(9)	74	67	128

*OS* overall survival, *S* surgery, *R* radiation, *C* chemotherapy, *RT* radiotherapy, *NA* data not available

In the present study, local recurrence was not observed in patients treated with PORT after surgical resection over a long follow-up duration, despite residual primary nidus after R1 resection in 11 (84.6%) patients. This clearly supports the role of RT for controlling microscopic disease in tracheal ACC. In contrast, there were 6 (66.7%) cases of LP in the definitive RT group. Hence, surgical resection of the tumor may be important for local tumor control, even though the tracheal tumor cannot be completely removed.

Nevertheless, definitive RT remains important in unresectable patients. Two-thirds of our study patients in definitive group lived for >5 years after RT, and the 10-year OS was 22.2%. Considering that these cases had relatively locally advanced tumors (T2 in 8 and T4 in 1 patient), this result does not appear to be disappointing, and hence, RT could be considered as an effective modality for unresectable tracheal ACC. Three patients developed LP more than 5 years after RT, which indicates that long-term evaluation with an appropriate imaging study should be recommended in these cases.

There is an issue in appropriate RT dose for unresectable tracheal ACC, but there is no consensus. In this study, patients treated with higher RT dose via BT boost had better long-term local tumor control and survival than those of patients receiving relatively lower RT dose. A 5 yr.-OS in patients treated with higher RT dose was very high, 88.3%. A 5 yr.-local control was 100% in 6 patients with higher RT dose, while all 3 patients with lower RT dose suffered from LP within 5 years after treatment (Fig. 1). Although this result was obtained from small number of patients (*n* = 9), it is sufficient to explain the necessity of escalated RT dose to sterilize gross tracheal ACC. We expect the IMRT can be a troubleshooter for escalating RT dose without additional treatment-related side effect, even if we used BT boost in most patients of this study that was done in period prior to clinical application of IMRT.

About 50% of ACC patients might eventually develop distant metastases, although regional lymph node involvement is reported to have been rare in several cohorts [6, 18]. In our present study, the main pattern of failure was distant metastasis. No regional lymph node metastasis was observed before or after treatment in our cases, but distant metastasis occurred in 63.6% of patients; the lung was the main metastatic site. The time interval between treatment and distant metastasis ranged from 8.4 to 91 months. Although a patient may develop a distant metastasis, they could remain alive with the metastasis for a long duration (up to 232 months); this may be related to the slow progression of ACC. The mean time interval from DM to death was 78 months, whereas the mean time interval between LP and death was 23 months in our series. This finding suggests that active local control can increase survival, although there is a risk of minimal distant metastasis in patients with tracheal ACC.

More than half of the patients in our present study lived for >5 years after definitive RT. Although this survival rate is inferior to that of the PORT group, it is sufficient to offer a feasible solution for patients with unresectable tracheal ACC. Nevertheless, LP developed between 5 years and 10 years after RT in 3 cases, and hence, the presence of symptoms in the airway should be monitored and proper imaging studies should be conducted for at least 10 years. The 10-year LPFS rate decreased to 26.7% in the present study cohort, and none of our patients exhibited LP after 10 years.

After resection with/without RT for tracheal cancer, some patients have been reported to develop severe complications such as recurrent laryngeal nerve palsy, tracheal stenosis, dysphagia, and airway granulation [6, 19–21]. In the present study, 2 patients developed tracheal stenosis, which resulted in death. Hence, the risk of tracheal stenosis should be carefully considered when determining the RT plan or dose in both PORT or definitive RT. Moreover, we should consider a suitable intervention for resolving the symptoms of stenosis, including the growth of granulation tissue [22].

In the current study, we enrolled a small number of patients over a period of 14 years, and analyzed the data retrospectively. However, we followed up patients over a mean period of 10 years and primarily assessed them based on the use of RT. Hence, we could readily determine that patients with unresectable tracheal ACC could survive for a long duration after definitive RT.

In summary, tumor stage and surgical excision might be important factor for survival or local tumor control. However, considering the high possibility of residual disease after surgical resection, postoperative adjuvant RT should be considered to sterilize microscopic tumors after surgery for tracheal ACC. Moreover, unresectable tracheal ACC can effectively be treated with definitive radiotherapy, which yields relatively long-term survival (>5 years) in most patients. Especially in patients with high-dose RT, definitive RT can warrant long-term survival and local tumor control over 5 years with proper methods for beam delivery like IMRT or BT. The major pattern of failure was lung metastasis, regardless of whether surgery was performed. However, tracheal stenosis remains a major concern after RT, and the RT plan or dose should accordingly be adjusted in some patients.

## Conclusion

Adjuvant radiotherapy for controlling microscopic residual disease in ACC may be suitable in cases of surgically resected tumors, whereas definitive radiotherapy may yield long-term survival in more than 50% of patients with unresectable tracheal ACC. Dose escalation should be considered to warrant long-term survival in definitive RT.

## Abbreviations

ACC: Adenoid Cystic Carcinoma
BT: Brachytherapy
C: Chemotherapy
CCRT: Concurrent Chemo-Radiotherapy
CR: Complete Remission
CT: Computed Tomography
DFS: Disease Free Survival
DMFS: Distant Metastasis Free Survival
EBRT: External Beam Radiotherapy
Gy: Gray
LP: Local Progression
LPFS: Local Progression Free Survival
mo: months
NA: data not available
OS: Overall Survival
PORT: Postoperative Radiotherapy
PR: Partial Remission
R: Radiation
R0: Complete Resection
R1: Microscopically Incomplete Resection
RESP: Initial response after RT
RT: Radiotherapy
RT-DM: Time interval from RT to DM
RT-LP: Time interval from RT to LP
S: Surgery
SCC: Squamous Cell Carcinoma
